# Microbial Basis for Suppression of Soil-Borne Disease in Crop Rotation

**DOI:** 10.3390/microorganisms12112290

**Published:** 2024-11-11

**Authors:** Boxi Wang, Shuichi Sugiyama

**Affiliations:** 1 Guangdong Engineering Laboratory of Biomass Value-Added Utilization, Institute of Biological and Medical Engineering, Guangdong Academy of Sciences, Guangzhou 510316, China; bessycc@163.com; 2 Faculty of Agriculture and Life Science, Hirosaki University, Hirosaki 036-8561, Japan

**Keywords:** Bacillales, clubroot, community network, disease resistance, Rhizobiales, soil conditioning, soil legacy effect, sustainable agriculture

## Abstract

The effect of crop rotation on soil-borne diseases is a representative case of plant–soil feedback in the sense that plant disease resistance is influenced by soils with different cultivation histories. This study examined the microbial mechanisms inducing the differences in the clubroot (caused by *Plasmodiophora brassicae* pathogen) damage of Chinese cabbage (*Brassica rapa* subsp. *pekinensis*) after the cultivation of different preceding crops. It addresses two key questions in crop rotation: changes in the soil bacterial community induced by the cultivation of different plants and the microbial mechanisms responsible for the disease-suppressive capacity of Chinese cabbage. Twenty preceding crops from different plant families showed significant differences in the disease damage, pathogen density, and bacterial community composition of the host plant. Structural equation modelling revealed that the relative abundance of four key bacterial orders in Chinese cabbage roots can explain 85% and 70% of the total variation in pathogen density and disease damage, respectively. Notably, the relative dominance of Bacillales and Rhizobiales, which have a trade-off relationship, exhibited predominant effects on pathogen density and disease damage. The disease-suppressive soil legacy effects of preceding crops are reflected in compositional changes in key bacterial orders, which are intensified by the bacterial community network.

## 1. Introduction

Species diversity in plant communities contributes to the inhibition of host-specific pathogen outbreaks and the resulting suppression of disease damage [1,2]. However, agricultural fields that aim for high yields under low diversity often suffer from severe disease damage without heavy use of agrochemicals. Crop rotation, that is, temporal changes in crop planting in identical fields, is known to be an effective method for suppressing soil-borne diseases by increasing temporal plant diversity [3,4], and it is essential for low-input sustainable agriculture.

Disease suppression by crop rotation is influenced by several factors including climatic conditions, cropping systems, and biotic and abiotic soil properties [3,4,5,6]. Among them, the identity of the preceding crop plays an important role in the suppression of soil-borne diseases. Since different plants harbour distinct microbial communities in the rhizosphere and root tissues even under identical soil environments [7,8,9,10], changes in the microbial community caused by different preceding crops influence the disease-suppressing capacity [3,6,11].

The effect of crop rotation on disease suppression can be viewed as a case of plant-soil feedback (PSF), i.e., the plant performance is influenced by soils with different cultivation histories. PSF consists of two processes: the conditioning phase, wherein biotic and abiotic soil properties are altered by plants, and the feedback phase, wherein changes in soil properties influence plant performance [12,13]. These two PSF processes result from complicated belowground interactions between plants and soil biota, including pathogens [14].

**Figure 1:**
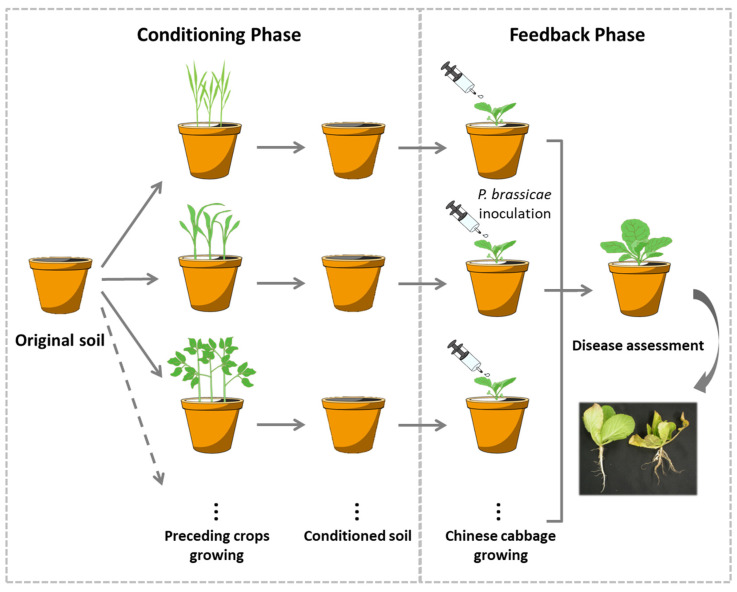
Schematic design of the experiment based on plant–soil feedback, in which 20 preceding crops were used for soil conditioning, which were individually planted in pots filled with identical soil, and then Chinese cabbage seedlings were raised in the conditioned soil that was inoculated with the clubroot pathogen.

In this study, we established an experimental system under the framework of PSF, in which 20 preceding crops belonging to different plant families were grown in single pots (i.e., the soil conditioning phase), and then Chinese cabbage (*Brassica rapa* subsp. *pekinensis*) seedlings were cultivated in the conditioned soils that were inoculated with the clubroot pathogen (i.e., the feedback phase), as shown in Figure 1. Pathogen abundance in Chinese cabbage roots was monitored using q-PCR, and bacterial communities in the soil and Chinese cabbage roots were analysed using the Illumina MiSeq platform [15]. In this experimental design, microbial processes involved in disease suppression can be traced back to the differences in preceding crops because a comparison is made between identical soils.

Plant disease damage is determined by the interactions between two forces: (1) the intensity of pathogen attack and (2) the protection of host plants against the attack [16,17]. Both forces are strongly influenced by the surrounding microbial community, which is characterised by tremendous diversity and a complex community network [18,19]. In contrast, specific microbial taxa can restrict pathogen growth by secretion of antibiotics and competitive inhibition and can activate the immune responses of host plants [15]. Two properties of a microbial community, the microbial consortia and network [20], and a limited number of key microbial taxa as suppressive agents [6,15] have been suggested to play important roles in disease suppression. However, it remains unclear as to which microbial properties play a major role in disease suppression and its components. In this study, we examined bacterial effects on the disease suppression of host plants through the analysis of its components, the restriction of pathogen growth in the roots, and other factors, including plant immune response, by evaluating the contributions of the two properties of the microbial community, i.e., diversity and network characterising the system and specific key taxa serving as suppression agents.

The central question of this study was how soil microbes mediate feedback between plants and soils. This requires an understanding of the microbial processes occurring during the conditioning and feedback phases. We addressed the following key questions of PSF: (1) What are the microbial changes responsible for disease suppression during the conditioning phase and (2) what are the microbial mechanisms causing the suppression of clubroot damage in the feedback phase? This is the first detailed study to analyse microbial processes that mediate PSF.

## 2. Materials and Methods

### 2.1. Experimental Design

The experiment was designed based on the PSF framework. The original well-mixed homogenous soils were cultivated (conditioned) with different plants (preceding crops), and Chinese cabbage seedlings were cultivated in conditioned soils inoculated with the clubroot pathogen (*Plasmodiophora brassicae*), as shown in Figure 1.

### 2.2. Soil and Plant Materials

The original soil used in the experiment was collected from the organic farming plot at the Hirosaki University farm (40°35′22.9″ N 140°28′20.8″ E, Hirosaki City, Aomori Prefecture, Japan), which had been managed without chemical fertilisers and agrochemicals for more than 5 years. The soil was classified as volcanic ash, with soil pH (water extraction) of 5.7, organic carbon (C) content of 7.89%, organic nitrogen (N) content of 0.59%, C/N ratio of 13.44, microbial C content of 64.53 mg per 100 g soil, and available phosphorous content of 3.0 mg per 100 g soil. The soils were sampled from the 0 to 10 cm layer and processed by sieving (2 mm mesh) to remove roots, macrofauna, and rocks; subsequently, they were used as the original soil in the experiment. A portion of each soil sample was stored at −80 °C for DNA analysis of the soil microbial communities.

To broaden the taxonomic background of the preceding crops, 20 plant species were selected from 20 different plant families belonging to 13 orders and two classes (monocot and dicot), which spanned an extensive range of angiosperms. The selected species were as follows: water spinach (*Ipomoea aquatica*), tomato (*Solanum lycopersicum*), perilla (*Perilla frutescens*), snapdragon (*Antirrhinum majus*), Canadian honewort (*Cryptotaenia canadensis*), crown daisy (*Glebionis coronaria*), platycodon (*Platycodon grandiflorus*), garden balsam (*Impatiens balsamina*), buckwheat (*Fagopyrum esculentum*), creeping spinach (*Basella alba*), plumed cockscomb (*Celosia argentea*), fringed pink (*Dianthus superbus*), spinach (*Spinacia oleracea*), cucumber (*Cucumis sativus*), soybean (*Glycine max*), okra (*Abelmoschus esculentus*), Chinese cabbage (*Brassica rapa*), corn poppy (*Papaver rhoeas*), Welsh onion (*Allium fistulosum*), and maize (*Zea mays*). Detailed information is provided in Table 1. Plant seeds were surface-sterilized using 1% sodium hypochlorite solution and germinated under sterile conditions. After one week of incubation at 22 °C and with a 16:8 h (day/night) photoperiod, five germinated seeds of each species were sown at equidistant positions in each pot (113 mm and 184 mm in diameter and height, respectively) and then transferred to an unheated glasshouse. Five replicates (pots) were used for each species. A total of 105 pots, including 5 control pots that did not contain any preceding crops, were established during the soil-conditioning phase (Figure 1). The preceding crops, including all belowground plant parts, were harvested 7–8 weeks after sowing depending on the growth rate of each species. The soil attached to the roots was removed to a feasible extent, and the soils were returned to their respective pots.

### 2.3. Chinese Cabbage Cultivation and Clubroot Disease Inoculation

Chinese cabbage seeds were surface-sterilized using 1% sodium hypochlorite solution for 20 min, washed three times in sterile distilled water (SDW) 3 times, and sown on sterile substrate media in Petri dishes. The seeds with Petri dishes were placed in a growth chamber at 22 °C with a photoperiod of 16:8 h (day/night) for 1 week. Four Chinese cabbage seedlings were transplanted into each of the 100 pots (113 mm and 184 mm in diameter and height, respectively) with conditioned soil and cultivated in an unheated glasshouse for 5 weeks. A nutrient solution (KNO_3_ 0.51 g/L+ KH_2_PO_4_ 0.14 g/L, 30 mL/pot) was applied in the 1st week after transplanting.

Clubroot gall was obtained from clubroot-infected cabbage (*Brassica oleracea*) grown on a farm in Hirosaki City. To prepare the resting spore suspension of *P. brassicae*, clubroot gall tissues (3 g) were immersed in 50 mL of SDW for 2 h for softening. The clubroot gall tissues were homogenised and filtered through eight layers of gauze. The filtrate was centrifuged at 1056× *g* rpm for 15 min, and the pellet was recovered and diluted with SDW to obtain a spore suspension. The concentration of the suspension was calibrated to 10^7^ mL^−1^ by a microscopy haemocytometer. The surface of the conditioned soil in each pot was inoculated with a spore suspension (30 mL) immediately after transplantation of the Chinese cabbage seedlings.

### 2.4. Sampling and Disease Assessment

Harvest and disease assessments were conducted at four developmental stages, starting at week 3 after sowing and at weekly intervals (i.e., weeks 3, 4, 5, and 6). At each harvest, one whole plant with attached soil was carefully harvested from each pot, without damaging the other plants. Five plants from each treatment (i.e., conditioned by the same preceding crop) were sampled and transferred to the laboratory. The plant samples were gently shaken to remove the maximum amount of attached soil, washed with tap water, and visually assessed.

The disease severity index (DSI) was used to evaluate disease damage [21], which was scaled as follows: no symptoms (0), galls only formed on the fibrous root (1), small galls formed on the lateral roots (3), galls formed on the lateral roots or small galls formed on the main root (5), many large galls formed on the lateral roots or galls formed on the main root (7), and severe galls formed on the main root, leading to partial degradation and the plant being nearly or already dead (9).

### 2.5. Sample Preparation and DNA Extraction

The root samples obtained from disease damage evaluation were sonicated in SDW at 50–60 Hz for 5 min (Ultrasonic Cleaner US-1, AS ONE, Osaka, Japan) to disrupt the tiny soil aggregates and attached microbes. Root samples were stored at −80 °C until processing. Five replicates of root and soil samples from each treatment (preceding crop condition) at each harvest were mixed with equivalent weights per replicate, and two technical replicates were prepared for each species for subsequent DNA extraction and Illumina sequencing. Root DNA was extracted using a DNAeasy Plant Mini Kit (Qiagen, Valencia, CA, USA). Soil DNA was extracted using bead-beating (Micro Smash MS-100; Tomy Seiko Co., Tokyo, Japan) and the ISOIL for Beads Beating Kit (Nippon Gene Co., Tokyo, Japan). The resulting DNA samples were assessed using a NanoDrop 2000 spectrophotometer (Thermo Fisher Scientific, Tokyo, Japan).

### 2.6. Quantification of P. brassicae Pathogen

To determine the density of *P. brassicae* in Chinese cabbage root tissues, the DNA of root samples at weeks 3 and 4 after inoculation was used for quantitative PCR (qPCR). The analysis was conducted using a 10 μL reaction volume and quantified using a DNA Engine Peltier Thermal Cycler (BioRad, Hercules, CA, USA) with six replicates per sample. The qPCR conditions were as follows: initial step at 95 °C for 10 min; 44 cycles at 95 °C for 15 s, 58 °C for 30 s, and 72 °C for 30 s; and final step at 72 °C for 5 min. qPCR was performed using *P. brassicae*-specific primers Pb4-1 (5′-TACCATACCCAGGGCGATT-3′) and PbITS6 (5′-CAACGAGTCAGCTTGAATGC-3′) [22].

### 2.7. PCR Amplification and Next-Generation Sequencing

We detected the microbiome structures of 20 preceding crop roots, original soil, conditioned soil, and Chinese cabbage roots sampled at week 3 (Figure 1). For the bacterial community, the V4 region of the 16S ribosomal RNA (rRNA) was amplified using the primer pair 515F (5′-GTGCCAGCMGCCGCGGTAA-3′) and 806R (5′-GGACTACHVGGGTWTCTAAT-3′) with adaptors in the 1st PCR amplification. To block chloroplast and mitochondrial amplification, peptide nucleic acid (PNA) clamps were included in the reaction [23]. The appropriate PNA concentration was determined by qPCR, and 2.5 μM of mPNA (mitochondria) and pPNA (plastid) were used in the 1st PCR amplification. The 1st PCR conditions were as follows: initial denaturation at 95 °C for 45 s; 35 cycles of denaturation at 95 °C for 15 s, PNA annealing at 78 °C for 10 s, primer annealing at 50 °C for 30 s, and extension at 72 °C for 30 s.

The 1st PCR products were purified using the Fast Gene Gel/PCR Extraction Kit (Nippon Genetic Co., Ltd., Tokyo, Japan) followed by the 2nd PCR with the primer pair, 2nd-F (5′-AATGATACGGCGACCACCGAGATCTACAC-Index-ACACTCTTTCCCTACACGACGC-3′) and 2nd-R (5′-CAAGCAGAAGACGGCATACGAGAT-Index’-GTGACTGGAGTTCAGACGTGTG-3′). The index pairs were specific for each sample to ensure accurate recognition of the samples. The 2nd 16S rRNA PCR conditions were as follows: initial denaturation at 94 °C for 2 min; 12 cycles at 94 °C for 30 s, 60 °C for 30 s, and 72 °C for 30 s; and a final extension at 72 °C for 5 min. The 2nd PCR products were purified using AMPureXP magnetic beads (Beckman-Coulter, Indianapolis, IN, USA) and pooled in equimolar ratios. After confirming the library quality, paired-end 2 × 250 bp sequencing of the barcoded amplicons was performed using a MiSeq sequencer (Illumina, San Diego, CA, USA).

### 2.8. Sequence Processing

The sequences obtained from MiSeq were processed using a custom pipeline developed by the Bioengineering Lab. Co., Ltd. (Atsugi, Japan). The raw reads were demultiplexed based on barcode sequences and filtered by exact matching using the Fastx toolkit (fastq_barcode_splilter). Reads with quality scores less than 20 and sequence lengths less than 40 bp were discarded. Paired-end reads with a minimum overlap of 10 bp were merged into full-length sequences using FLASH [24]. Merged sequences between 246 and 260 bp were used for subsequent processing of 16S rRNA. The UCHIME algorithm was used to detect the chimeric sequences [25]. Operational taxonomic unit (OTU) generation and phylogenetic assignment were conducted using QIIME. The OTUs were clustered using UCLUST [26] at a 97% similarity level with a de novo picking method and the Greengenes 16S reference database [27]. All OTUs assigned to archaea, chloroplasts, and mitochondria were discarded. The filtered OTU dataset was normalised by transforming OTU counts to their relative abundance. The complete dataset was deposited in the DDBJ Sequence Read Archive (DRA) database (DRA accession: DRA008315 for root microbiomes of 20 plant families and DRA009770 for soil microbiomes and root microbiomes of Chinese cabbage). Spurious sequences and unrepresentative OTUs decrease the reproducibility of community assemblage. Illumina sequence analysis of soil and root bacterial communities, including the roots of 20 preceding crops, provided 6,148,077 total reads that were assigned to 367,240 OTUs. To increase data reproducibility, we set a threshold level of 0.06% based on the correlation between Log10-transformed relative abundance in the same OTU in technical replicates (Figure S1) [8]. Low-abundance OTUs below this level were omitted from the analysis; therefore, the number of OTUs declined to 1299, and the total number of reads used was reduced by 4,815,725 (78.3%).

### 2.9. Statistical Analysis and Visualization

Two-way ANOVA was used to detect the differences among the four developmental stages and the 20 preceding crop treatments for *P. brassicae* pathogen density (PD) and DSI. Differences between the preceding crop treatments at each developmental stage were also tested using one-way ANOVA and Tukey–Kramer HSD for multiple comparisons. PD was ln-transformed to improve normality and homoscedasticity. The Shannon–Weaver index and the number of observed OTUs were calculated using the vegan package in R (v3.1.1) [28] to evaluate the α-diversity of the microbial communities. The Bray–Curtis distance dissimilarity matrix, which is a representation of β-diversity, was calculated using the vegdist function in the vegan package. Principal coordinate analysis (PCoA) was performed using the Bray–Curtis distance dissimilarity with the APE package in R [29]. Network analysis was performed to determine the relationships among the bacterial taxa, PD, and DSI using the CoNet app in Cytoscape (v3.8.0). Pearson’s correlation coefficient was calculated using JMP (v4.05J, SAS. Cary, NC, USA).

Linear discriminant analysis Effect Size (LEfSe, v1.0) was used to identify biomarkers characterising different groups [30]. The non-parametric factorial Kruskal–Wallis sum-rank test was performed to detect significant differences in abundance between groups (class). The resulting subset of vectors was used to build a Linear Discriminant Analysis (LDA) model. The alpha-value for both the Kruskal–Wallis and Wilcoxon tests was 0.05, and the logarithmic LDA threshold score for discriminative features was 2.

Structural equation modelling (SEM) was conducted using AMOS 18 (SPSS, Chicago, IL, USA) to test the direct and indirect effects of each bacterial taxon on the suppression of clubroot damage in Chinese cabbage. In the model, a direct effect represents a link with the DSI, whereas an indirect effect represents a link through PD. In the initial model, all the direct and indirect links of bacterial orders that showed significant correlations with PD and DSI were built. The goodness of fit of the model was assessed using the χ^2^ value and Goodness of Fit Index (GFI). Links were removed from the model when the χ^2^ value decreased and the GFI increased. This process was repeated until the model reached its maximum goodness-of-fit value. The degree of freedom, χ^2^ value, and GFI in the final mode (which included four bacterial orders) were 12, 5.567, and 0.917, respectively, compared with 53, 108.79, and 0.503, respectively, in the initial model.

To determine the soil conditioning effects on the composition of bacterial communities, we analysed the pairwise correlations of the relative abundances of the important bacterial orders in each of the conditioned soil and Chinese cabbage root communities, and between the two communities. A path analysis was also conducted using AMOS, excluding one anomalous value in the conditioned soil.

## 3. Results

### 3.1. Effects of Preceding Crops on Clubroot Damage

Clubroot damage in the infected Chinese cabbage roots, represented by DSI, showed a substantial increase with the progression of developmental stages (Figure S2). Both developmental stage and preceding crop treatment, as well as their interactions, were the main factors explaining the variations in DSI (*p* < 0.001, Table S1). Although the DSI did not show a significant difference among the preceding crops in week 3, significant differences (*p* < 0.001) became apparent in week 4 (Table S2). However, significant differences disappeared in weeks 5 and 6 because most individuals had already exhibited severe damage (Figure S2, Table S2).

In week 4, heavy damage was observed in the plants raised in soils conditioned by the two monocot species (DSI of 77.8 for maize and 73.3 for Welsh onion), whereas minor damage was observed in those conditioned by Canadian honewort, platycodon, and soybean (DSI of 11.1, 24.4, and 26.7, respectively), as shown in Table 1. These results demonstrate that the taxonomic identity of the preceding crops plays an important role in the suppression of clubroot damage.

Significant positive correlations were detected between DSI and PD (ln-transformed). PD at week 3 showed a more significant positive correlation with DSI than that at week 4 (*r* = 0.73, *p* < 0.001, Figure S3), suggesting that PD in the infected roots at a 1-week earlier stage was the critical factor in determining the difference in DSI. The PD at week 3 was the highest in the soils conditioned by maize and Welsh onion, which showed the most severe damage in Chinese cabbage roots, whereas soils conditioned by Canadian honewort and platycodon with the lowest DSI had the lowest PD (Table 1).

### 3.2. Changes in Bacterial Communities Due to Soil Conditioning

Although the number of observed OTUs showed significant differences between the preceding crop treatments both in the soil and Chinese cabbage root (Table S3), no significant correlations were observed with PD and DSI both for the soil (*r* = 0.03 and *r* = −0.27) and the root communities (*r* = 0.08 and *r* = −0.01). Therefore, the OTU richness did not exhibit a clear relationship with disease suppression.

The relative abundance of the bacterial taxa was calculated to examine the effects of community composition on disease suppression. The taxonomic identification of each OTU was less apparent at deeper taxonomic levels. The taxonomic identification rates were 99.9%, 92.7%, 70.2%, 28.7%, and 3.0% at the class, order, family, genus, and species levels, respectively; thus, the order level was chosen because of its high identification rate. Therefore, 90 orders were obtained, and principal coordinate analysis (PCoA) was applied using their relative abundances.

**Figure 2:**
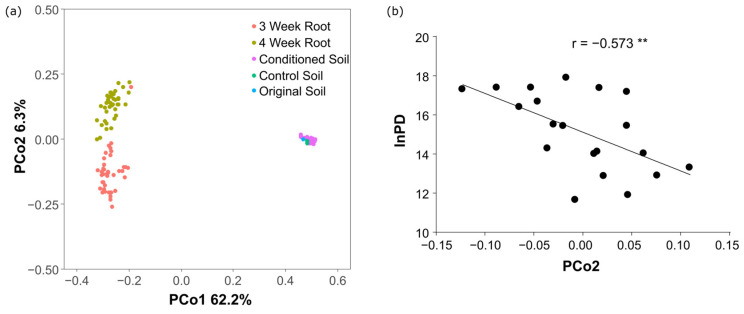
Plots depicting the principal coordinate analysis (PCoA) of soil and root bacterial communities among the 20 preceding crop treatments. (**a**) Scatter diagram of the first and second components in the soil (closed circles) and root communities (triangles), including the original soil before conditioning (Osoil, open circle). (**b**) Correlation between the score of the second component of the root bacterial community and PD. lnPD represents the ln-transformed copy number of pathogen density. ** *p* < 0.01.

The bacterial community composition showed large divergence between the soil and root communities across the first axis of the PCoA (Figure 2a). The soils conditioned by the 20 preceding crops did not show as great variations in microbial communities as of roots. The roots communities varied across the second axis, demonstrating a great effect of the preceding crops on the composition of the root microbial community. The scores of conditioned root samples in the second axis of PCoA (PCo2) were used as representations of the variations among the root communities. A significant correlation was detected between root PCoA scores and PD (*r* = −0.573, *p* < 0.01, Figure 2b) and a marginally significant correlation with DSI (*r* = −0.437). These results suggest that pathogen growth was dependent on the composition of the bacterial community in the Chinese cabbage roots.

**Figure 3:**
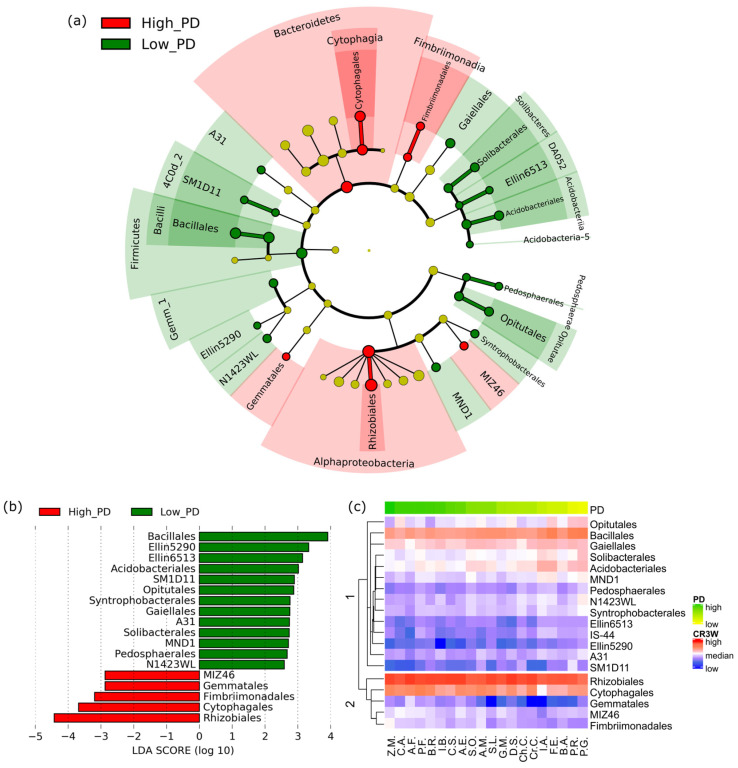
Linear discriminant analysis effect size (LEfSe) identifying biomarkers (microbial taxa) related to high- or low-PD and their enrichment patterns. (**a**) Cladogram representing the taxonomic distribution of biomarkers characterising high- and low-PD; (**b**) plot of the effect size of detected biomarkers represented by linear discriminant analysis (LDA) score; (**c**) heatmap of high- and low-PD abundant biomarkers. The preceding crop treatments were ordered by PD value, and the detected biomarkers were clustered according to Bray–Curtis distance dissimilarity.

Next, we performed linear discriminant analysis effect size (LEfSe) analysis to identify biomarkers on the phylogenetic tree that significantly influenced PD. A total of 20 preceding crop treatments were separated into high- and low-PD groups by the threshold of lnPD = 15; the high-PD group included maize, plumed cockscomb, Welsh onion, perilla, Chinese cabbage, and garden balsam, in which lnPD ranged from 17 to 18, and the low-PD group included corn poppy, platycodon, creeping spinach, and buckwheat, in which lnPD ranged from 11.6 to 12.9 (Table 1). Many bacterial groups were scattered across the phylogenetic tree, and bacterial taxa that discriminated between the two groups were identified (Figure 3a). The classes Alphaproteobacteria, Cytophagia, and Fimbriimonadia were identified as biomarkers of the high-PD group, while Bacilli, Optutae, and Acidobacteriia were biomarkers of the low-PD group (Figure 3). At the order level, 5 orders including Rhizobiales, Cytophagales, and Fimbriimonadales were identified as biomarkers of the high-PD group, and 13 orders including Bacillales, Ellin5290, and Ellin6513 were identified as those of the low-PD group (Figure 3a,b). Rhizobiales and Bacilalles were the most prominent orders, characterised by high- and low-PD groups, respectively. The heatmap was generated, revealing the overall pattern of relative abundances of these detected orders among PD value changes across the 20 preceding crops. The bacterial orders clustered into two groups based on Bray–Curtis dissimilarity (Figure 3c). The composition of clusters 1 and 2 confirmed the low-PD and high-PD biomarker clarification from the LEfSe results (Figure 3a,b).

### 3.3. Microbial Basis for Suppression of Clubroot Damage

**Figure 4:**
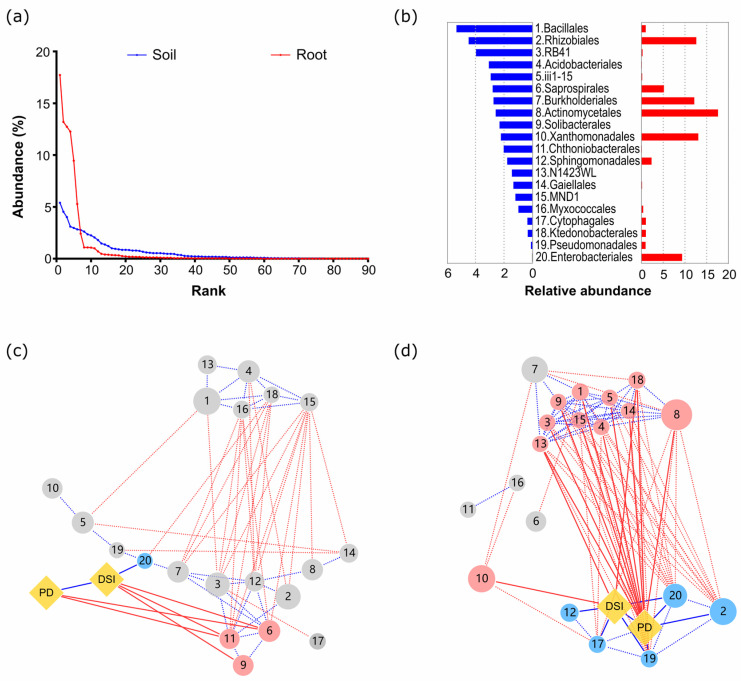
Rank–abundance curve and the correlation network among the top 20 bacterial orders, PD and DSI. Rank–abundance curve of 90 bacterial orders in the soil (blue) and root (red) microbial communities (**a**) and the relative abundances of the top 20 orders in the soil (blue) and root (red) bacterial communities (**b**). The numbers in networks of the soil (**c**) and root communities (**d**) refer to (**b**). The size of each circle represents relative abundance. The blue, red, and grey circles represent the co-present, mutually exclusive, and neutral groups in terms of their relationships with PD, respectively. The lines show the presence of significant correlation. The red and blue lines between the circles indicate the presence of significantly negative and positive correlations, respectively. Solid lines indicate the correlations with PD and DSI, and dotted lines indicate correlations between bacterial orders.

A total of 90 orders used for the PCoA analysis showed a disproportionate contribution of the relative abundances to the total share according to their abundance rank: a small number of top-ranked orders had an extremely high contribution, and the contribution exponentially decreased with their rank order (Figure 4a). For example, the top 4 orders in the root community, which are Actynomycetales (17.7%), Rhizobiales (13.2%), Burkholderiales (12.7%), and Xanthomonodales (12.3%), shared more than 10% of the proportion, and the sum of the top 20 orders reached 79.2% of the total share. The top 20 orders in the soil community shared 42.7% of the total, and the abundances of Bacillales and Rhizobilales were only 5.4% and 4.5%, respectively (Figure 4b). We focused on the top 20 high-abundance orders in the subsequent analysis.

Network analysis revealed that connectedness among the top 20 orders differed significantly between the soil and root communities (Figure 4c,d). The density and the average number of edges per node in the network were 0.381 and 7.62 for the root community and 0.157 and 3.76 for the soil community, respectively, indicating that a more closely connected network developed in the root community. High connectedness in the root community was also supported by a close connection with PD.

**Figure 5:**
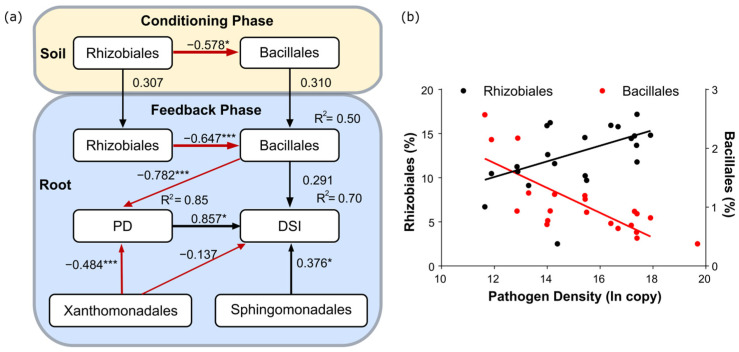
(**a**) Schematic representation of structural equation modelling (SEM) explaining the microbial basis of clubroot suppression during the conditioning and feedback phases. The model at the feedback phase indicated that the key bacterial order (Bacillales) and two other orders (Xanthomonodales and Sphingomonadales) explained 70% of the variation in clubroot damage (DSI) and 85% of the variation in PD. The model at the conditioning phase showed that the abundance differences in Bacillales in the Chinese cabbage roots were mainly the result of a weak colonisation effect from the soil to the root and the enhancement of the initial difference by a strong antagonistic relationship with Rhizobiales. The values of the arrows represent the standardised regression coefficient. (**b**) Plot depicting the relationships between PD and the relative abundance of Bacillales (red, *r* = −0.747 ***) and Rhizobiales (black, *r* = 0.623 **). lnPD represents the ln-transformed copy number of pathogen density. * *p* < 0.05; ** *p* < 0.01; *** *p* < 0.001.

Structural equation modelling (SEM) was used to clarify the complicated relationships between bacterial taxa and disease suppression in the roots. The initial model consisted of ten orders that showed significant correlations with PD and/or DSI (Figure S4). Among them, eight orders had significant correlations with PD and three orders had significant correlations with DSI. The best-fit SEM model selected four bacterial orders (Bacillales, Rhizobiales, Xanthomonodales, and Sphingomonadales) and explained 85% of the total variation in the PD and 70% of the variation in the DSI (Figure 5a). The effects of Bacillales and Xanthomonadales on PD were highly significant (standardised regression coefficients of −0.782, *p* < 0.01 and −0.484, *p* < 0.05, respectively), whereas a significant coefficient for DSI was only found for Sphingomonadales (0.376 *).

Among the 20 bacterial orders, Bacillales and Rhizobiales were the key bacterial taxa responsible for the suppression of pathogen growth, as indicated by their significant negative (*r* = −0.747, *p* < 0.01) and positive (*r* = 0.623, *p* < 0.01) correlations with PD, respectively (Figure 5b). These significant correlations at the order level were the result of many correlations at the deeper taxonomic levels (genera). In Bacillales, all eight genera showed negative correlations with PD, and these correlations were significant in three genera. In contrast, six genera in Rhizobiales showed significant positive correlations with PD (Table 2).

### 3.4. Legacy Effects of Preceding Crops

**Figure 6:**
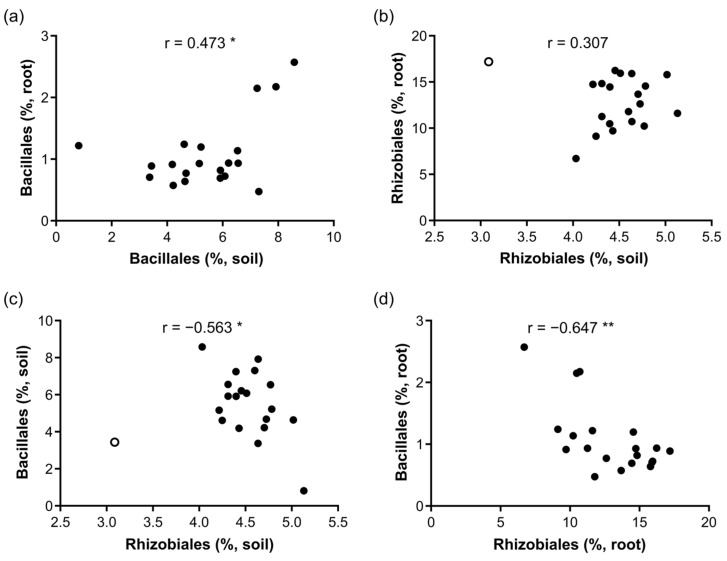
Plots showing the relationships between the relative abundances of the soil and root communities for Bacillales (**a**) and Rhizobiales (**b**), and the relationships between the abundances of the two orders in the soil (**c**) and root communities (**d**). Open circles represent anomalous samples of Rhizobiales in the soil community, which were omitted from the analysis. * *p* < 0.05; ** *p* < 0.01.

The results demonstrate the importance of the composition of the root bacterial community in determining disease suppression. The differences in the root bacterial community can be traced back to changes in the soil community during soil conditioning by the preceding crops. Thus, we examined the compositional changes from the soil to the root communities in terms of both the whole community structure and the abundance of the specific key taxon. The structure of the entire community was characterised by PCoA based on 90 bacterial orders, as shown in Figure 2. The PCoA scores of the second axis did not show a significant correlation between the root and soil communities (*r* = 0.08), indicating that the entire community structure did not succeed from the conditioned soils to Chinese cabbage roots. Contrarily, the abundance of the two key taxa, Bacillales and Rhizobiales, showed weak positive correlations between the conditioned soils and Chinese cabbage roots, a significantly positive correlation (*r* = 0.473, *p* < 0.05) for Bacillales, and a weak positive correlation (*r* = 0.307) for Rhizobiales (Figure 6). These results indicate that the abundance of the two key taxa of Chinese cabbage roots partly succeeded from the conditioned soil to the Chinese cabbage roots.

The abundance of Bacillales in the Chinese cabbage root was explained only up to 22% by the regression with the abundance in the soil, whereas the proportion increased to 50% when the abundance of Rhizobiales in both the soil and the root was included in the model (Figure 5a). The results demonstrate that the presence of a strong antagonist (Rhizobiales) enhanced the abundance of Bacillales in the Chinese cabbage roots (Figure 5a). Furthermore, the presence of a network connection in the entire community in the Chinese cabbage root, as shown in Figure 4, can intensify the roles of key suppressive agents.

## 4. Discussion

### 4.1. Microbial Basis for Disease Suppression

Field observations have shown that the density of resting spores of clubroot in soils is one of the most important factors determining the differences in clubroot damage [31,32,33]. In this study, PD had the greatest effect on clubroot damage in Chinese cabbage. Despite the tremendous diversity of the soil bacterial community, a single bacterial order, Bacillales, had a predominant effect on PD, as shown in Figure 5a. Among the eight genera belonging to Bacillales, one genus, *Bacillus*, showed a highly negative correlation with PD (*r* = −0.77, *p* < 0.01, Table 2) and is known to suppress clubroot damage [5,34]. *Bacillus* can exclude other bacterial cells, including pathogens, after colonising the root surface [35]. Thus, it is used as a biofungicide to suppress clubroot damage under field conditions. However, the relative abundance of Bacillales in Chinese cabbage roots ranging from 0.5 to 2.6% is not likely to be high enough to cause competitive inhibition of pathogen growth. The suppressive effects of *Bacillus* on colonisation and proliferation of clubroot pathogens may be due to complicated mechanisms, including the secretion of antibiotics and activation of immune response of host plants.

This study also showed the high contribution of Rhizobiales to disease suppression. However, Rhizobiales acted negatively in terms of disease suppression, i.e., an increase in clubroot pathogens. Rhizobiales had a relatively high abundance in the Chinese cabbage roots, ranging from 6.7 to 17%, and 19 genera showed significant positive correlations with PD (Table 2). These results imply that the increased abundance of Rhizobiales in Chinese cabbage roots produces conditions suitable for the growth of clubroot pathogens. Contrarily, the members of Rhizobiales reportedly perform important functions, such as nitrogen-fixing and methanotrophy [36,37] and, thus, confer beneficial functions to host plants, such as providing nutrients and phytohormones and improving soil productivity [38,39,40]. The negative effects of Rhizobiales on the disease susceptibility of host plants may be cancelled out by its positive effects on the growth promotion of host plants.

The relative dominance of Bacillales and Rhizobiales in Chinese cabbage roots was modulated by the preceding crops. As in preceding crops, Asterales and Apiales enhanced the disease suppression of Chinese cabbage by increasing the abundance of Bacillales, while two families belonging to the monocot caused great damage to Chinese cabbage roots by increasing the abundance of Rhizobiales. These results confirm earlier field observations that clubroot damage is alleviated more by using soybean as a preceding crop than by using cereal [41]. These results also suggest that endophytic bacteria living in the roots exhibit phylogenetic divergence with respect to the effects of host plant functions.

In addition to Bacillales and Rhizobiales, other bacterial orders were significantly correlated with PD in the Chinese cabbage roots. Enterobacteria, Ktedonobacteriales, Cytophagales, and Pseudomonadales, which have been reported to be associated with disease suppression in soil [18,20], were also significantly correlated with PD (Figure 4d). However, the contribution of these bacterial orders to PD was masked by the close correlation network, including Bacillales and Rhizobiales, in the SEM model. This correlation network among bacterial taxa in Chinese cabbage roots may play an important role in determining the disease suppression of host plants, as discussed below.

The immune responses of host plants contribute to disease resistance [16,17]. Although the SEM model explained 70% of the variation in the disease suppression (DSI) of Chinese cabbage among the 20 preceding crop treatments, most were explained by PD. Therefore, the restriction of pathogen growth seems to be more important in the suppression of clubroot damage than the activation of the host immune response. However, since Bacillales, Sphingomonadales, and Xanthomonadales directly contribute to DSI, as shown in Figure 5a, disease-suppressive mechanisms other than the restriction of pathogen growth should be involved in the suppression of clubroot damage.

### 4.2. Legacy Effects of Conditioned Soil on Disease Suppression

The difference in the abundance of key bacterial taxa, such as Bacillales and Rhizobiales, in Chinese cabbage roots originated from the conditioning phase, in which 20 preceding crops were cultivated in identical soils. However, the weak correlation between the abundance of the conditioned soil and that of Chinese cabbage roots (Figure 6) revealed that the abundance differences in the Chinese cabbage roots did not directly reflect the differences in the conditioned soil. The abundance of Rhizobiales and Bacillales in Chinese cabbage root was a result of the following: (1) the colonisation process from the soil to the root and (2) subsequent biological interactions among colonised microbes in root tissues. Microbe colonisation from the soil to the roots is subject to stochastic processes. Contrastingly, the strong positive or negative correlations among bacterial taxa in the Chinese cabbage roots contributed to the enhancement of initial abundances produced during the colonisation process. The negative correlation between Bacillales and Rhizobiales, reflecting biological interaction in the root, was stronger than that of the correlation between the soil and root communities, representing the colonisation effect. Specifically, the stochastic abundance variation occurring during the colonisation process is strongly organized by a deterministic process of interactions among bacterial taxa inside the root.

This study shows the importance of a small number of key bacterial taxa in the suppression of clubroot damage. Contrarily, the study demonstrates the importance of the community networks among many bacterial taxa. For example, the abundance of Bacillales in the Chinese cabbage roots merely explained 36% of the variation in PD, whereas the proportion increased to 85% when three other orders were included in the model. These results demonstrate that a community network consisting of many bacterial taxa supports the importance of a small number of suppressive agents.

## 5. Conclusions

This study reveals that the complicated microbial processes induced by crop rotation contribute to the suppression of soil-borne diseases. The processes based on plant–soil feedback involve complex interactions among host plants, microbes, and pathogens, such as the preferential selection of specific microbial taxa by host plants and the spontaneous development of community networks among bacterial taxa in roots. Because of this complexity, the effects of crop rotation on disease suppression in fields are likely to differ depending on the soil conditions, microbial community structure, and target plants. The specification of key bacterial taxa in this study may be attributed to the simplicity of the experimental design, wherein differences in the background soil microbiome were accounted for by the use of identical soils. Therefore, further understanding of the assembly rules of crop root microbiomes and their effects on host crop functions under agricultural field conditions (including different soil types, different host crops, and a high degree of environmental heterogeneity) is required for the development of low-input sustainable agriculture.

## Figures and Tables

**Table 1:** Clubroot disease severity index (DSI) and pathogen density (PD) of Chinese cabbage roots. The scientific name and abbreviation of 20 different preceding crops are listed.

No.	Abbreviation	Preceding Crops	Scientific Name	DSI (SE)	lnPD (SE)
1	Z.M.	Maize	*Zea mays* subsp. *mays*	77.8 (0.00)	17.9 (0.16)
2	A.F.	Welsh onion	*Allium fistulosum*	73.3 (8.31)	17.4 (0.25)
3	S.O.	Spinach	*Spinacia oleracea*	73.3 (4.44)	15.5 (0.18)
4	P.F.	Perilla	*Perilla frutescens*	64.4 (8.89)	17.4 (0.34)
5	S.L.	Tomato	*Solanum lycopersicum*	60.0 (4.44)	15.4 (0.42)
6	A.M.	Snapdragon	*Antirrhinum majus*	55.6 (0.00)	15.4 (0.08)
7	A.E.	Okra	*Abelmoschus esculentus*	55.6 (7.03)	16.4 (0.25)
8	B.R.	Chinese cabbage	*Brassica rapa* subsp. *pekinensis*	55.6 (9.94)	17.3 (1.91)
9	C.A.	Plumed cockscomb	*Celosia argentea*	51.1 (4.44)	17.4 (0.17)
10	C.S.	Cucumber	*Cucumis sativus*	48.9 (12.96)	16.7 (0.11)
11	I.B.	Garden balsam	*Impatiens balsamina*	42.2 (5.44)	17.2 (0.09)
12	B.A.	Creeping spinach	*Basella alba*	37.8 (8.31)	12.9 (2.14)
13	I.A.	Water spinach	*Ipomoea aquatica*	37.8 (4.44)	13.3 (0.25)
14	F.E.	Buckwheat	*Fagopyrum esculentum*	37.8 (4.44)	12.9 (0.58)
15	D.S.	Fringed pink	*Dianthus superbus*	37.8 (4.44)	14.1 (0.84)
16	P.R.	Corn poppy	*Papaver rhoeas*	33.3 (7.03)	11.9 (2.41)
17	Ch.C.	Crown daisy	*Chrysanthemum coronarium*	28.9 (4.44)	14.0 (0.20)
18	G.M.	Soybean	*Glycine max*	26.7 (9.69)	14.3 (0.08)
19	P.G.	Platycodon	*Platycodon grandiflorus*	24.4 (5.44)	11.6 (0.07)
20	Cr.C.	Canadian honewort	*Cryptotaenia canadensis*	11.1 (0.00)	14.0 (0.13)
Significance				*p* < 0.001	*p* < 0.001

lnPD represent the ln-transformed copy number of pathogen density.

**Table 2:** Taxonomic identity of OTUs belonging to Bacillales and Rhizobilales at the genus level and their correlations with ln-transformed PD in the root and DSI. * *p* < 0.05; ** *p* < 0.01; *** *p* < 0.001.

Order	Family	Genus	DSI (*p*)		PD (*p*)	
Bacillales	Paenibacillaceae	*Cohnella*	0.01		−0.19	
	Paenibacillaceae	*Ammoniphilus*	0.04		−0.29	
	Paenibacillaceae	*Brevibacillus*	−0.16		−0.32	
	Staphylococcaceae	*Staphylococcus*	−0.17		−0.22	
	Paenibacillaceae	*Paenibacillus*	0.19		−0.14	
	Planococcaceae	*Solibacillus*	−0.22		−0.52	*
	Alicyclobacillaceae	*Alicyclobacillus*	−0.28		−0.49	*
	Bacillaceae	*Bacillus*	−0.45	*	−0.77	***
Rhizobiales	Bradyrhizobiaceae	*Balneimonas*	0.16		0.38	
	Bradyrhizobiaceae	*Bosea*	−0.31		−0.55	*
	Brucellaceae	*Ochrobactrum*	−0.33		−0.15	
	Hyphomicrobiaceae	*Devosia*	0.50	*	0.64	**
	Hyphomicrobiaceae	*Hyphomicrobium*	0.25		0.09	
	Hyphomicrobiaceae	*Pedomicrobium*	−0.07		−0.18	
	Hyphomicrobiaceae	*Rhodoplanes*	0.04		−0.19	
	Methylobacteriaceae	*Methylobacterium*	−0.39		−0.22	
	Phyllobacteriaceae	*Mesorhizobium*	0.60	**	0.69	***
	Rhizobiaceae	*Agrobacterium*	0.48	*	0.72	***
	Rhizobiaceae	*Kaistia*	0.42		0.71	***
	Rhizobiaceae	*Rhizobium*	−0.02		0.25	
	Rhodobiaceae	*Afifella*	0.47	*	0.46	*
	Xanthobacteraceae	*Labrys*	0.54	*	0.58	**

## Data Availability

The raw reads of sequences were deposited into the DDBJ Sequence Read Archive (DRA) database (DRA accession: DRA008315 for root microbiomes of 20 plant families and DRA009770 for soil microbiomes and root microbiomes of Chinese cabbage).

## Supplementary Materials

The following supporting information can be downloaded at: https://www.mdpi.com/article/10.3390/microorganisms12112290/s1, Figure S1. The relationship between Log10-transformed relative abundances of the two technical replicates in each OTU. The red line represents the threshold level (0.06%) used in this study; Figure S2. (**a**) Changes in means of disease severity index (DSI) of Chinese cabbage roots during the 3rd to 6th weeks after the infection of clubroot pathogen. (**b**) Frequency distribution of DSI during the 3rd to 6th week; Figure S3. The relationship between pathogen density (PD) ln-transformed copy number of clubroot in the third week and disease severity index (DSI); Figure S4. The initial model of structural equation modelling using 10 bacterial orders in the root communities that showed significant correlations with PD or DSI. The black and red arrows are positive and negative correlations, and the values side of the arrow are correlation coefficients. The χ^2^ value and GFI in the model were 108.79 and 0.503, respectively; Table S1. α-diversity (mean values) of soil and Chinese cabbage root microbial communities. The variances between samples were tested by one-way ANOVA; the variances of the interaction between the development stage and pre-crop species were tested by two-way ANOVA (* *p* < 0.05; ** *p* < 0.01; *** *p* < 0.001); Table S2. Analysis of variance for disease damage index (DSI). Variances of development stage, pre-crop species, and interaction on DSI by two-way ANOVA; Table S3. Analysis of variance for DSI among preceding crop treatments at each developmental stage. Variances of pre-crop species on DSI at each development stage by one-way ANOVA.
