# Supplementary material for: Microbial Basis for Suppression of Soil-Borne Disease in Crop Rotation
Source: Microorganisms. 2024 Nov 11;12(11):2290. doi: 10.3390/microorganisms12112290 (PMC11596175; doi:10.3390/microorganisms12112290)
Supplement: Supplementary file 1 [file microorganisms-12-02290-s001.zip › microorganisms-3292967-supplementary.pdf]

Supplementary

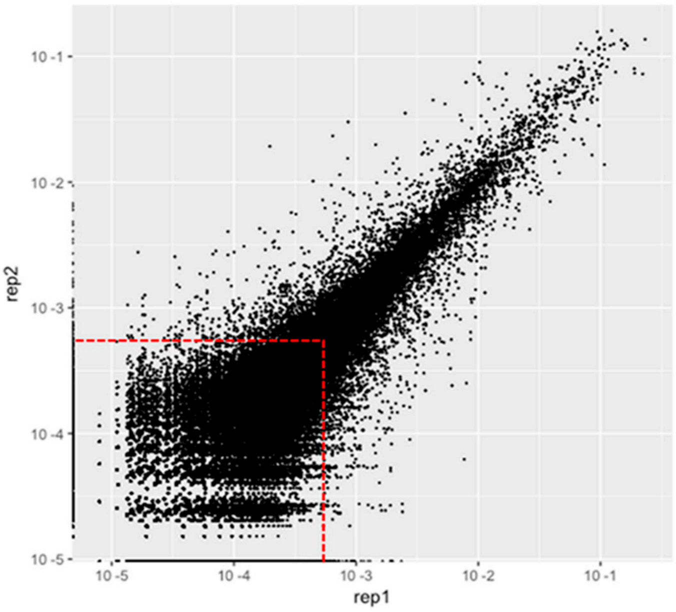

Figure S1. The relationship between Log^{10}-transformed relative abundances of the two technical replicates in each OTU. The red line represents the threshold level (0.06%) used in this study.

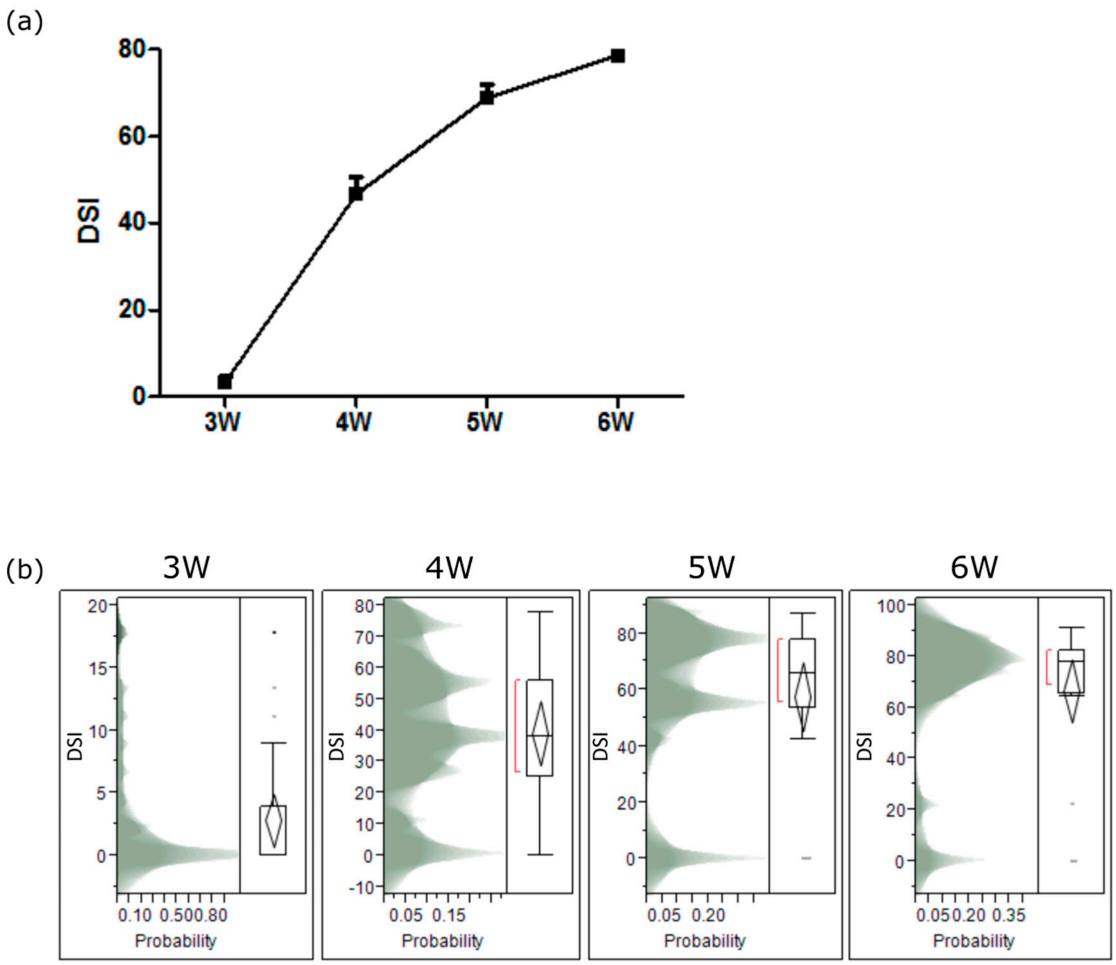

Figure S2. (**a**) Changes in means of disease severity index (DSI) of Chinese cabbage roots during the 3rd to 6th weeks after the infection of clubroot pathogen. (**b**) Frequency distribution of DSI during the 3rd to 6th week.

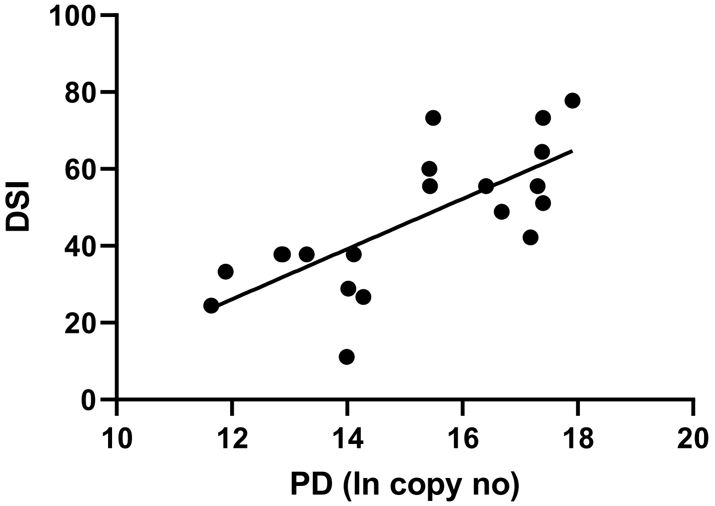

Figure S3. The relationship between pathogen density (PD) ln-transformed copy number of clubroot in the third week and disease severity index (DSI).

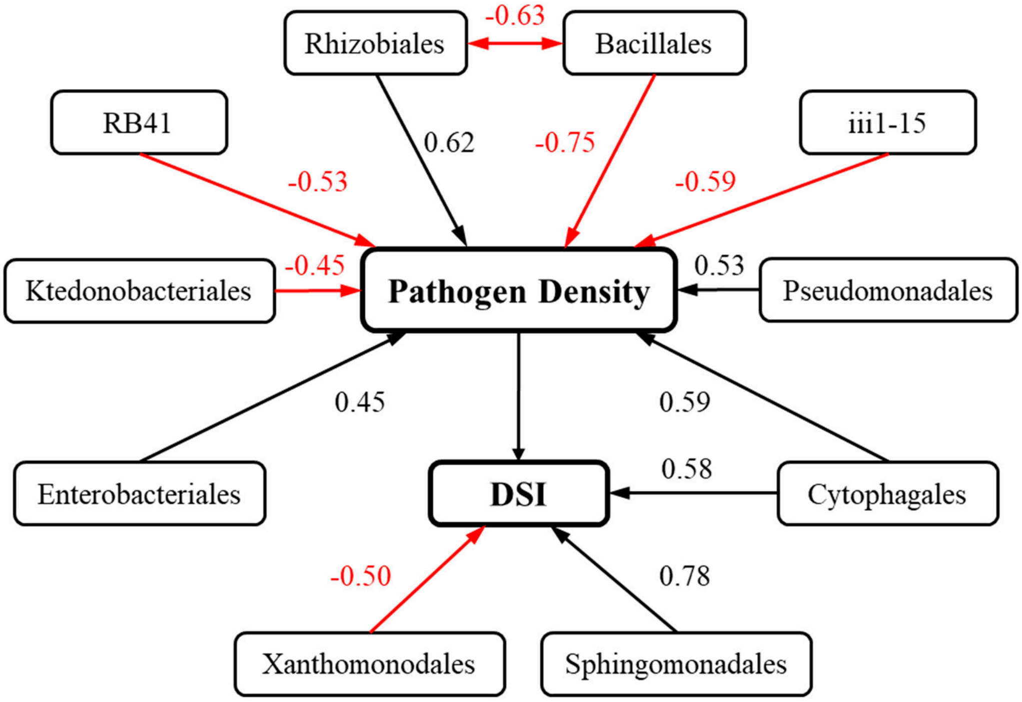

Figure S4. The initial model of structural equation modelling using 10 bacterial orders in the root communities that showed significant correlations with PD or DSI. The black and red arrows are positive and negative correlations, and the values side of the arrow was correlation coefficients. The $\chi 2$ value and GFI in the model were 108.79 and 0.503, respectively.

Table S1. α-diversity (mean values) of soil and Chinese cabbage root microbial communities. The variances between samples were tested by one-way ANOVA; the variances of the interaction between the development stage and pre-crop species were tested by two-way ANOVA (* $p < 0.05$; ** $p < 0.01$; *** $p < 0.001$).

	Shannon index	Observed OTUs
CS	7.39	8289
CR	5.39	6227
Pr(>F)	<0.001 ***	<0.001 ***
CR3rd week	5.35	6823
CR4th week	5.44	5631
Pr(>F)	0.07	<0.001 ***

Table S2. Analysis of variance for disease damage index (DSI). Variances of development stage, pre-crop species and interaction on DSI by two-way ANOVA.

**Factors**	**df**	**MS**	**F**	**Pr(>F)**
**Stage (S)**	3	111857.92	381.09	<0.001
**Preceding crops (P)**	19	1288.16	4.39	<0.001
**Interaction (SxP)**	57	549.50	1.87	0.0004
**Error**	320	293.52		

Table S3. Analysis of variance for DSI among preceding crop treatments at each developmental stage. Variances of pre-crop species on DSI at each development stage by one-way ANOVA.

**Stage**	**F**	**Pr (>F)**
**3 week**	1.182	0.293
**4 week**	7.386	<0.001
**5 week**	1.483	0.115
**6 week**	1.246	0.244
